# Thermal Management and Modeling of Forced Convection and Entropy Generation in a Vented Cavity by Simultaneous Use of a Curved Porous Layer and Magnetic Field

**DOI:** 10.3390/e23020152

**Published:** 2021-01-26

**Authors:** Fatih Selimefendigil, Hakan F. Öztop

**Affiliations:** 1 Department of Mechanical Engineering, Celal Bayar University, Manisa 45140, Turkey; fatih.selimefendigil@cbu.edu.tr; 2 Department of Mechanical Engineering, Technology Faculty, Fırat University, Elazığ 23119, Turkey; 3 Department of Medical Research, China Medical University Hospital, China Medical University, Taichung 40402, Taiwan

**Keywords:** curved porous layer, vented cavity, convection, finite volume method, nanofluid, entropy generation

## Abstract

The effects of using a partly curved porous layer on the thermal management and entropy generation features are studied in a ventilated cavity filled with hybrid nanofluid under the effects of inclined magnetic field by using finite volume method. This study is performed for the range of pertinent parameters of Reynolds number (100≤Re≤1000), magnetic field strength (0≤Ha≤80), permeability of porous region ($10^{-4}\leq \mathrm{Da}\leq 5\times 10^{-2}$), porous layer height ($0.15\;H\leq t_{p}\leq 0.45H$), porous layer position ($0.25\;H\leq y_{p}\leq 0.45\;H$), and curvature size (0≤b≤0.3H). The magnetic field reduces the vortex size, while the average Nusselt number of hot walls increases for Ha number above 20 and highest enhancement is 47% for left vertical wall. The variation in the average Nu with permeability of the layer is about 12.5% and 21% for left and right vertical walls, respectively, while these amounts are 12.5% and 32.5% when the location of the porous layer changes. The entropy generation increases with Hartmann number above 20, while there is 22% increase in the entropy generation for the case at the highest magnetic field. The porous layer height reduced the entropy generation for domain above it and it give the highest contribution to the overall entropy generation. When location of the curved porous layer is varied, the highest variation of entropy generation is attained for the domain below it while the lowest value is obtained at $y_{p}=0.3\;H$. When the size of elliptic curvature is varied, the overall entropy generation decreases from b = 0 to b=0.2H by about 10% and then increases by 5% from b=0.2H to b=0.3H.

## 1. Introduction

Convective heat transfer (CHT) in vented cavities is relevant in a variety of different technological applications such as in electronic cooling, textiles, drying, heating-ventilation, air conditioning, and many other systems [1,2,3,4]. Layered porous structures are considered in various applications such as as in fuel cell, solidification, and many other numerous systems. In heat transfer applications, porous inserts may be used for CHT control in channel flow or they may be already available within the system. In the study of Chikh et al. [5], thickness of the porous layer was found to be very effective on the CHT coefficient for the partly porous channels. In the study of Siavashi et al. [6], gradient and layered porous foams were utilized for the performance improvement of CHT in a channel, while best condition of multi-layered foams was obtained by using an optimization routine. In the study of Guerroudj and Kahalerras [7], shape effects of porous blocks were analyzed for mixed CHT and profound impacts of the shape on the heat transfer were reported. Free CHT in a layered domain of nanofluid and porous media was explored in the work of Miroshnichenko et al. [8]. It was noted that the porous media thickness and location were effective on the CHT features. Astanina et al. [9] explored the the effects of porous layer on the CHT a lid-driven cavity. They noted that the average Nu reduced for higher thickness of porous layer.

Magnetic field (MF) effects are explored for the CHT in the vented cavity. The MF effects are relevant in diverse technological applications such as in nuclear reactor coolers, geothermal energy, micro-pumps [10], blood flow through arteries [11], and many other numerous applications in convective heat transfer. In many CHT applications, MF effects were reported to reduce the CHT rate, while in configurations where flow separation exist, MF effects may be opposite due to the suppression of the flow recirculations. Binary particles of nanosized Ag/MgO are suspended in water which is considered as the heat transfer fluid (HTF). Nanofluid technology is successfully implemented in many heat transfer system including many renewable energy stems and CHT [12,13]. Over the years, many different simulation methods have been developed to correctly describe the nanofluid behavior along with the new correlations for effective nanofluid property relations. Hybrid nanofluids are considered in many heat transfer applications due to the cost, stability and favorable features of thermophysical properties [14,15].

MF effects can be used effectively by utilizing nanoparticles in the HTF. The thermal conductivity enhances by using nanofluid and on the other hand, the electrical conductivity changes as well which will have impacts when MF effects are present. Many studies are considered which take into account the MF effects with nanofluid for CHT control [16,17,18].

Second law analysis is also performed for various geometric and operating parameters of the thermo-fluid system. The system performance and optimal working conditions can be assessed by utilization of the second law. Minimization of the entropy generation concept has been developed in thermal processes and many factors that were found to be effective in the entropy generation have been explored [19]. In many studies of CHT, entropy generation and exergy loss analysis have been performed by many authors for the case of MF and nanofluid effects [20,21,22].

In the present study, forced CHT and entropy generation in a VC are numerically explored by using a partly curved porous layer under the impacts of inclined MF by using hybrid nanofluid. The nanofluid thermophysical properties are available from the experimental study. To the best of the authors’ knowledge, simultaneous use of a curved porous layer with MF effects has never been considered in VC for thermal management in the cavity. Owing to diverse use of CHT in vented cavities in various technological applications, the results of this novel thermo-fluid configuration will be used to develop new designs and for further optimization studies.

## 2. Mathematical Modeling

### 2.1. Physical Problem

**Figure 1:**
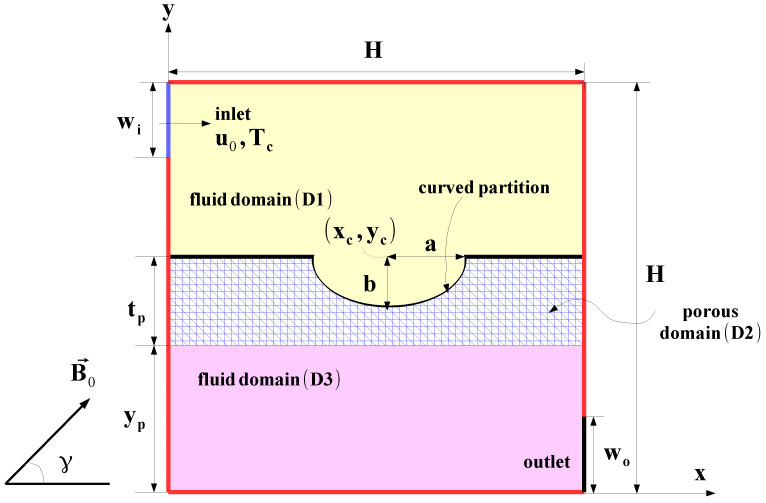
Schematic view of the considered transfer fluid (TF) configuration with curved porous layer.

A ventilated cavity (VC) with one input and one output port is considered as shown in Figure 1. The size of the ports is $w_{i}=w_{o}=0.25\;H$ with *H* being the cavity height. In the VC, a curved porous layer is introduced below the inlet port with thickness of $t_{p}$, while $y_{p}$ is the vertical distance between the left bottom corner of VC. An elliptic-shaped curved interface between the porous and fluid layers at center location $\left(x_{c},y_{c}\right)$ with radii of *a* and *b* is included. Fluid with velocity of u${}_{0}$ and T${}_{c}$ cold temperature enter the inlet, and the walls of the VC are at T${}_{h}$ hot temperature. Hybrid nanofluid with Ag and MgO particles is used. A uniform magnetic field (MF) is imposed in nanofluid and porous regions and inclination angle of MF is considered as γ=45°. Natural convection effects with viscous dissipation and radiation and are not considered while the induced MF effects and joule heating are ignored. The flow is 2D, steady, and in the laminar regime while fluid is Newtonian.

In the nanofluid regions (D1 and D3), conservation equations (CEs) are stated as [22,23]

(1) $$\frac{\partial u}{\partial x}+\frac{\partial v}{\partial y}=0$$

(2) $$\begin{array}{cc} \; & \left(u\frac{\partial u}{\partial x}+v\frac{\partial u}{\partial y}\right)=-\frac{1}{\rho_{nf}}\frac{\partial p}{\partial x}+\nu_{nf}\left(\nabla^{2}u\right)\\ \; & +\frac{\sigma_{nf}B_{0}^{2}}{\rho_{nf}}\left(v\sin\left(\gamma \right)\cos\left(\gamma \right)-u\sin^{2}\gamma \right) \end{array}$$

(3) $$\begin{array}{cc} \; & \left(u\frac{\partial v}{\partial x}+v\frac{\partial v}{\partial y}\right)=-\frac{1}{\rho_{nf}}\frac{\partial p}{\partial y}+\nu_{nf}\left(\nabla^{2}v\right)\\ \; & +\frac{\sigma_{nf}B_{0}^{2}}{\rho_{nf}}\left(u\sin\left(\gamma \right)\cos\left(\gamma \right)-v\cos^{2}\gamma \right) \end{array}$$

(4) $$u\frac{\partial T}{\partial x}+v\frac{\partial T}{\partial y}=\alpha_{nf}\nabla^{2}T.$$

In the porous region (domain D2), the generalized Darcy-Brinkmann Forchheimer model was considered with the CEs [23]:

(5) $$\frac{\partial u}{\partial x}+\frac{\partial v}{\partial y}=0$$

(6) $$\begin{array}{cc} \; & \frac{1}{\varepsilon^{2}}\left(u\frac{\partial u}{\partial x}+v\frac{\partial u}{\partial y}\right)=-\frac{1}{\rho_{nf}}\frac{\partial p}{\partial x}+\frac{\nu_{nf}}{\varepsilon }\left(\nabla^{2}u\right)\\ \; & -\nu_{nf}\frac{u}{K}-\frac{F_{c}}{\sqrt{K}}u\sqrt{u^{2}+v^{2}}+\frac{\sigma_{nf}B_{0}^{2}}{\rho_{nf}}\left(v\sin\left(\gamma \right)\cos\left(\gamma \right)-u\sin^{2}\gamma \right) \end{array}$$

(7) $$\begin{array}{cc} \; & \frac{1}{\varepsilon^{2}}\left(u\frac{\partial v}{\partial x}+v\frac{\partial v}{\partial y}\right)=-\frac{1}{\rho_{nf}}\frac{\partial p}{\partial y}+\frac{\nu_{nf}}{\varepsilon }\left(\nabla^{2}v\right)\\ \; & -\nu_{nf}\frac{v}{K}-\frac{F_{c}}{\sqrt{K}}u\sqrt{u^{2}+v^{2}}+\frac{\sigma_{nf}B_{0}^{2}}{\rho_{nf}}\left(u\sin\left(\gamma \right)\cos\left(\gamma \right)-v\cos^{2}\gamma \right) \end{array}$$

(8) $$u\frac{\partial T}{\partial x}+v\frac{\partial T}{\partial y}=\alpha_{nf}\nabla^{2}T.$$

Non-dimensional parameters are written as:

(9) X=xH,Y=yH,U=uv0,V=vv0,P=pρnfv02θ=T−TcTh−TcFc=1.75150ε2,Pr=νfαf,Re=v0Hnf,Ha=B0Hσnfρnff,Da=KH2

Non-dimensional CEs are stated as [22,23]

(10) $$\frac{\partial U}{\partial X}+\frac{\partial V}{\partial Y}=0$$

(11) 1(δ1ε)2U∂U∂X+V∂U∂Y=−∂P∂X+d11(δ1ε)Re∇2U−d1UDaReδ2−FcDaUU2+V2δ2+d2Ha2ReVsin(γ)cos(γ)−Usin2γ

(12) 1(δ1ε)2U∂V∂X+V∂V∂Y=−∂P∂Y+d11(δ1ε)Re∇2V−d1VDaReδ2−FcDaVU2+V2δ2+d2Ha2ReUsin(γ)cos(γ)−Vcos2γ

(13) U∂θ∂X+V∂θ∂Y=d31RePr∇2θ.

with $d_{1}=\frac{\nu_{nf}}{\nu_{f}}$, $d_{2}=\frac{\rho_{f}}{\rho_{nf}}\frac{\sigma_{nf}}{\sigma_{f}}$, and $d_{3}=\frac{\alpha_{nf}}{\alpha_{f}}$. Here, $\delta_{1}$ and $\delta_{2}$ take the value of 1 for domain D2, while $\delta_{1}=\frac{1}{\varepsilon }$ and $\delta_{2}=0$ for the domains D1 and D3.

Boundary conditions are stated as

- VC inlet: u = u${}_{0}$, v = 0, T = T${}_{c}$
- VC exit: $\frac{\partial u}{\partial x}=\frac{\partial T}{\partial x}=0,\;v=0$
- Interface between the layers: $u_{f}=u_{p},\;v_{f}=v_{p},k_{f}{\left(\frac{\partial T}{x}\right)}_{f}=k_{p}{\left(\frac{\partial T}{x}\right)}_{p}$
- VC walls: T = T${}_{c}$

Entropy generation includes the terms due to heat transfer, viscous dissipation, and magnetic field, and it can be written as [22]

(14) $$\begin{array}{c} S_{g}=\frac{k_{f}}{T_{0}^{2}}\left[{\left(\frac{\partial T}{\partial x}\right)}^{2}+{\left(\frac{\partial T}{\partial y}\right)}^{2}\right]+\frac{\mu }{T_{0}}\left[2\left({\left(\frac{\partial u}{\partial x}\right)}^{2}+{\left(\frac{\partial v}{\partial y}\right)}^{2}\right)+{\left(\frac{\partial u}{\partial x}+\frac{\partial v}{\partial y}\right)}^{2}\right]\\ +\frac{\sigma B_{0}^{2}}{T_{0}}{\left(u\sin\gamma -v\cos\gamma \right)}^{2} \end{array}$$

with $T_{0}=\frac{T_{c}+T_{h}}{2}$.

Nusselt numbers (local and average) are defined as

(15) $${\mathrm{Nu}}_{l}=-\frac{k_{nf}}{k_{f}}\left(\frac{\partial \theta }{\partial n}\right),\;\;{\mathrm{Nu}}_{m}=\frac{1}{L}\int_{0}^{L}{\mathrm{Nu}}_{l}dl.$$

where *L* is the total length for each of the individual hot walls of the VC.

As in the HTF, water with Ag-MgO binary nanoparticles is considered and Table 1 shows the various thermophysical properties [24].

The nanofluid density and specific heat are given as [24]

(16) $$\rho_{nf}=\left[\left(1-\phi_{2}\right)\left(\left(1-\phi_{1}\right)\rho_{f}+\phi_{1}\rho_{s1}\right)\right]+\phi_{2}\rho_{s2}$$

(17) $${\left(\rho c_{p}\right)}_{nf}=\left[\left(1-\phi_{2}\right)\left(\left(1-\phi_{1}\right){\left(\rho c_{p}\right)}_{f}+\phi_{1}{\left(\rho c_{p}\right)}_{s1}\right)\right]+\phi_{2}{\left(\rho c_{p}\right)}_{s2}$$

Experimental data were used for the description of the thermal conductivity and viscosity of the hybrid nanofluid.

Thermal conductivity is stated as [25]

(18) $$k_{nf}=\left(\frac{0.1747\times 10^{5}+\phi }{0.1747\times 10^{5}-0.1498\times 10^{6}\phi +0.1117\times 10^{7}\phi^{2}+0.1997\times 10^{8}\phi^{3}}\right)k_{f}$$

where ϕ is the total solid volume fraction of two different nanoparticles which is defined as:

(19) $$\phi =\phi_{1}+\phi_{2}.$$

Viscosity of nanofluid is given as [25]:

(20) $$\mu_{nf}=\left(1+32.795\phi -7214\phi^{2}+714600\phi^{3}-0.1941\times 10^{8}\phi^{4}\right)\mu_{f}.$$

Electrical conductivity of the hybrid nanofluid is defied by using the Maxwell relation as [26]

(21) $$\sigma_{nf}=\sigma_{f}\left(\frac{1+3\left(\frac{\sigma }{\sigma_{f}}-1\right)\left(\phi_{1}+\phi_{2}\right)}{\left(\frac{\sigma }{\sigma_{f}}+2\right)-\left(\frac{\sigma }{\sigma_{f}}-1\right)\left(\phi_{1}+\phi_{2}\right)}\right)$$

where σ denotes the following:

(22) $$\sigma =\frac{\sigma_{1}\phi_{1}+\sigma_{2}\phi_{2}}{\phi_{1}+\phi_{2}}.$$

Due to the lack of experimental correlation for the effective electrical conductivity of hybrid nanofluid containing Ag/MgO binary particles in water, the above model is preferred which was also used in the study in [27]. However, in the literature, different models that were derived from the experimental data were available for the electrical conductivity of nanofluid with several different nanoparticles. In the study of Selimefendigil and Öztop [28], effects of different electrical conductivity models for water–alumina nanofluids on the mixed convection features were explored. Minea and Luciu [29] performed experimental work for the electrical conductivity of Al${}_{2}$O${}_{3}$ nanofluids, and they developed a correlation for the effective electrical conductivity of nanofluid which dependent upon the solid volume fraction and temperature. A strong impact of the volume fraction was noted. In the experimental work of Chereches and Minea [30], electrical conductivity of hybrid nanofluids with water as base fluid and Al${}_{2}$O${}_{2}$, TiO${}_{2}$ and SiO${}_{2}$ as nanoparticles was examined for the temperature range of 20 °C and 60 °C. Several relations for the effective electrical conductivity of hybrid nanofluid were developed.

### 2.2. Solution Method and Code Validation

As the solution of the CEs with boundary conditions, the finite volume method (FVM) is utilized. A commercial computational fluid dynamics code based on FVM, Fluent [31], is used as the solver. After using the appropriate discretization schemes for diffusion and convective terms, the algebraic equations are obtained as [32]

(23) $$a_{p}\phi_{p}=\sum a_{n}\phi_{n}+s$$

where *p* and *n* are the node point and relevant neighbor node, respectively.

The QUICK (Quadratic Upstream Interpolation for Convective Kinematics) scheme is utilized for convective terms discretization [33] while SIMPLE (Semi-Implicit Method for Pressure Linked Equations) is selected for the velocity-pressure coupling [34]. The solution is made by using the Gauss-Siedel point-by-point iterative method and algebraic multigrid method [31]. The residual which is in normalized form is stated as

(24) $$R^{\phi }=\frac{\sum_{all\;cells}\left|a_{p}\phi_{p}-a_{n}\phi_{n}-s\right|}{\sum_{all\;cells}\left|a_{p}\phi_{p}\right|}.$$

Solution convergence is obtained for residual value less than 10${}^{-8}$ (for all dependent variables). Under-relaxation parameters are used, and they are taken as 0.6 for velocity and temperature.

**Figure 2:**
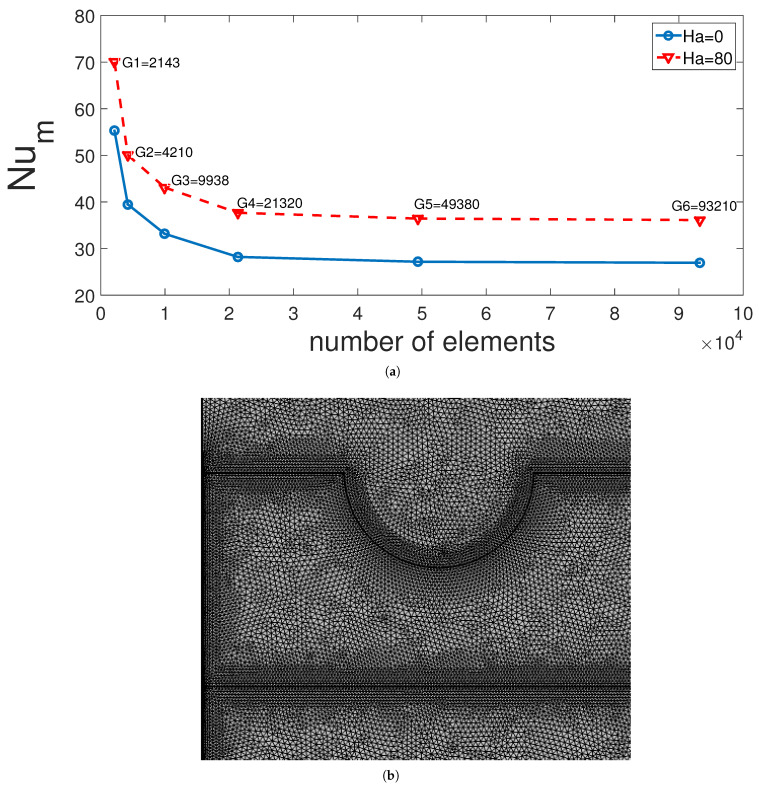
Grid independence results: The average Nu comparisons at two MF strength considering different grid sizes (**a**) and grid distribution (**b**).

Different grid sizes are tested for assurance of mesh independence of the solution. Figure 2a shows the average Nu considering all hot walls of VC at two different Hartmann numbers for various grids. G4 with 21,320 elements is selected, while the mesh distribution is given in Figure 2b. The mesh is refined near the walls and at the interface of domains D1 and D2, and D2 and D3.

**Figure 3:**
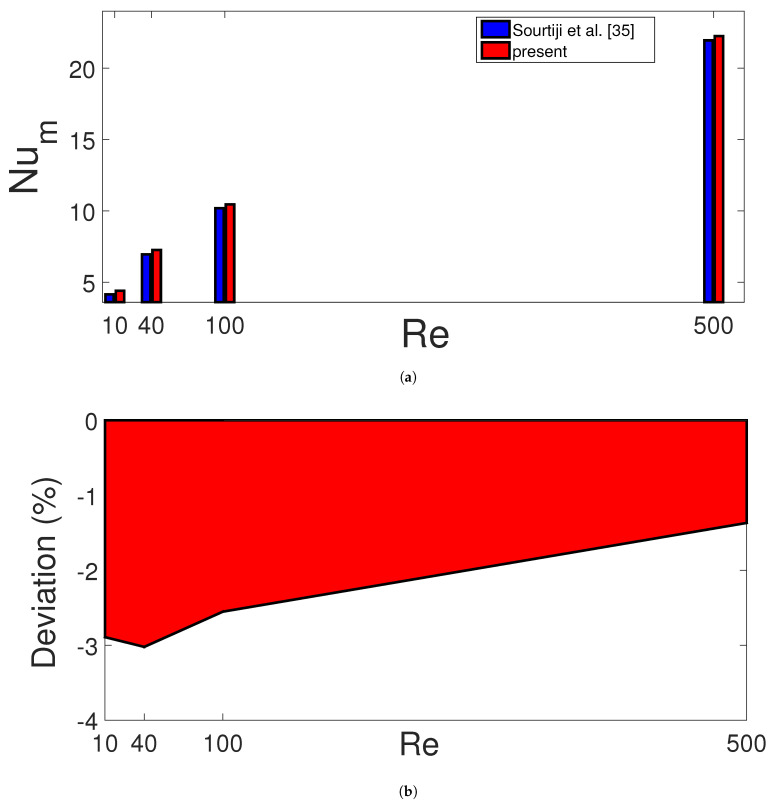
Comparison of average Nu for different Reynolds number at Ri = 10 (**a**) and deviation in percentage between the present work and reference study in [35] (**b**).

Code validation was performed. In the first work, numerical study results of [35] were used where CHT in a vented cavity was explored. Figure 3 shows the comparison results of average Nu for different Reynolds numbers while highest deviation below 3.5% is obtained. In another validation study, CHT in a porous cavity was considered. Table 2 shows the comparison results of average Nu values with different sources at two values of Rayleigh number while the agreement between the results seems satisfactory. The last validation is performed by using the numerical results in Ref. [36] where CHT in a cavity with magnetic field effects was considered. Table 3 presents the comparison results of average Nu for two different Rayleigh numbers at Hartmann number of 30. The highest deviation is below 4%.

## 3. Results and Discussion

Forced convective heat transfer (CHT) in a VC with a partly curved porous layer under uniform MF impacts are studied. Hybrid nanofluid is utilized as the HTF. The study is conducted for the pertinent parameters of Reynolds number (100≤Re≤1000), MF strength (0≤Ha≤80), permeability of porous region ($10^{-4}\leq \mathrm{Da}\leq 5\times 10^{-2}$), height of porous layer ($0.15H\leq t_{p}\leq 0.45\;H$), location of porous layer ($0.25\;H\leq y_{p}\leq 0.45\;H$), and elliptic curvature radius (0≤b≤0.3H). The other radius of the ellipse is taken as a=0.2H while the center location is chosen as $\left(x_{c},y_{c}\right)=\left(0.5\;H,y_{p}+t_{p}\right)$. The hybrid particles solid volume fraction is chosen as ϕ=2% while MF inclination angle is γ=45°.

**Figure 4:**
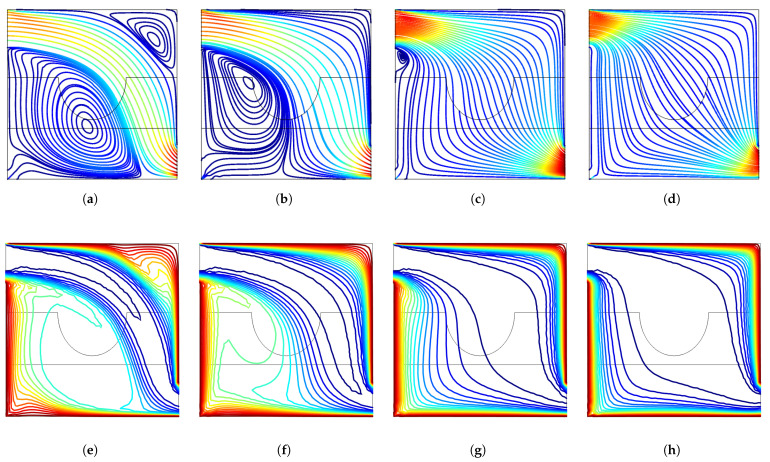
MF strength impacts on the variation of streamlines and isotherms (Re = 500, γ=45°, Da = $5\times 10^{-2}$, t${}_{p}$ = 0.3 H, y${}_{p}$ = 0.3 H, b = 0.25 H). (**a**) Ha = 0, (**b**) Ha = 20, (**c**) Ha = 40, (**d**) Ha = 80, (**e**) Ha = 0, (**f**) Ha = 20, (**g**) Ha = 40, (**h**) Ha = 80.

A uniform inclined MF is imposed in the computational domain of VC. In the absence of MF, recirculation zones are established below and above the main flow stream (Figure 4). The size of the vortex below the inlet is gradually reduced with higher MF strength while the vortex near the upper corner disappears with MF. The MF effects resulted in thinner thermal boundaries along the hot walls of the VC. The inclined MF is seen to suppress the recirculation zones within the VC.

**Figure 5:**
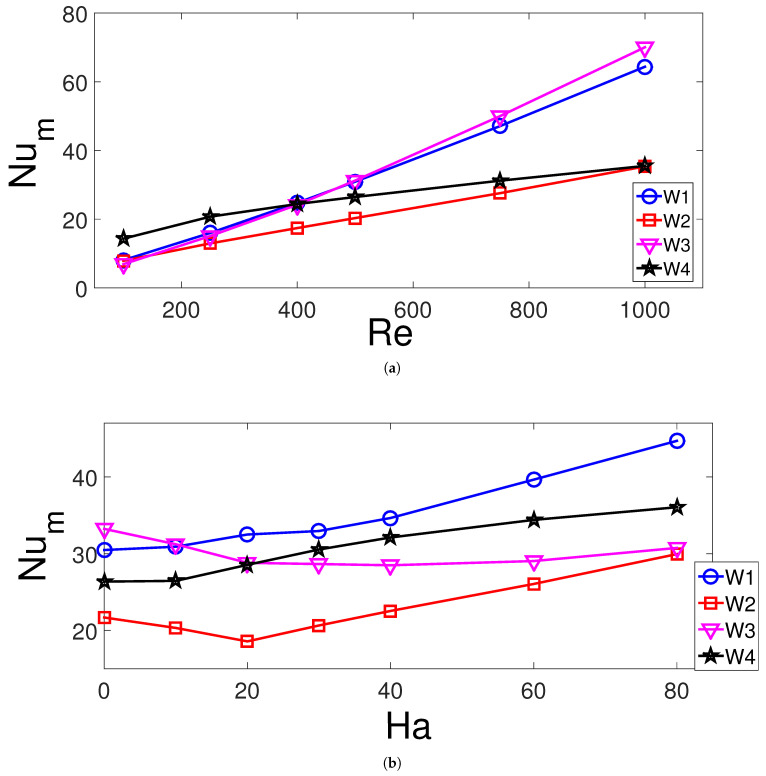
Effects of Re number and MF strength on the average Nu variation of hot walls (γ=45°, Da = $5\times 10^{-2}$, t${}_{p}$ = 0.3 H, y${}_{p}$ = 0.3 H, b = 0.25 H). (**a**) Ha = 10, (**b**) Re = 500.

Figure 5 presents the average Nu (Nu${}_{m}$) variation for each of the hot walls for varying Reynolds number and MF strength considering each of the hot walls of the VC. Here, W1, W2, W3, and W4 represent the left, bottom, right, and top hot walls, respectively. The Nu${}_{m}$ increases with higher Re, while the highest impact is seen for left and right walls of the VC. For the left hot wall, this is attributed to the vortex size reduction below inlet and more cold fluid interacts with the hot wall. As the MF strength is increased to Ha = 20, the Nu${}_{m}$ for bottom and left wall reduces and increases thereafter. The MF acts in away to rise the average Nu${}_{m}$ for other walls of the VC. The highest increment in the average Nu is seen for hot wall W1 (47%), and it is followed by walls W2 (38%) and W4 (38%) as the cases in the absence and presence of MF are compared. This could be attributed to the Lorentz forces of the MF, the vortex which is occurred below inlet port reduces with higher MF strength. However, the average Nu reduces by about 6.8% for wall W3.

**Figure 6:**
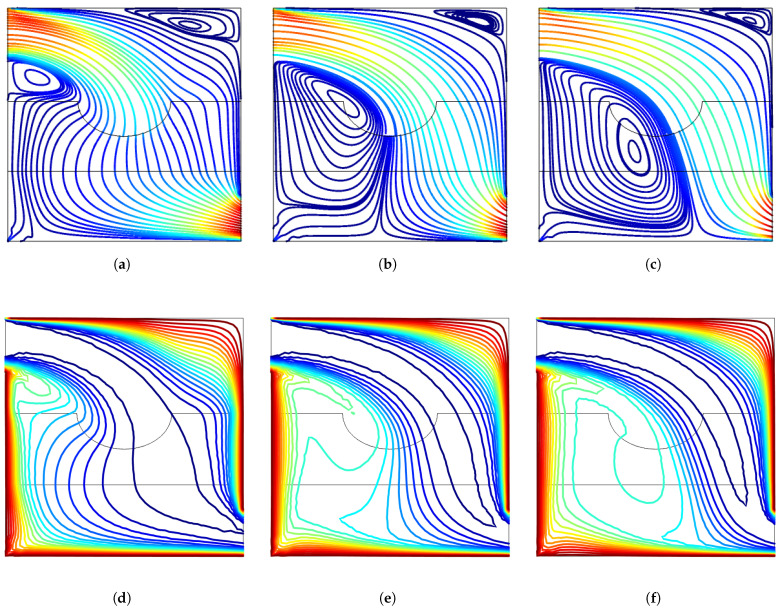
Impacts of porous layer permeability on the variation of streamlines and isotherms (Re = 500, Ha = 15, γ=45°, t${}_{p}$ = 0.3 H, y${}_{p}$ = 0.3 H, b = 0.25 H). (**a**) Da = 10${}^{-4}$, (**b**) Da = 10${}^{-3}$, (**c**) Da = $5\times 10^{-2}$, (**d**) Da = 10${}^{-4}$, (**e**) Da = 10${}^{-3}$, (**f**) Da = $5\times 10^{-2}$.

**Figure 7:**
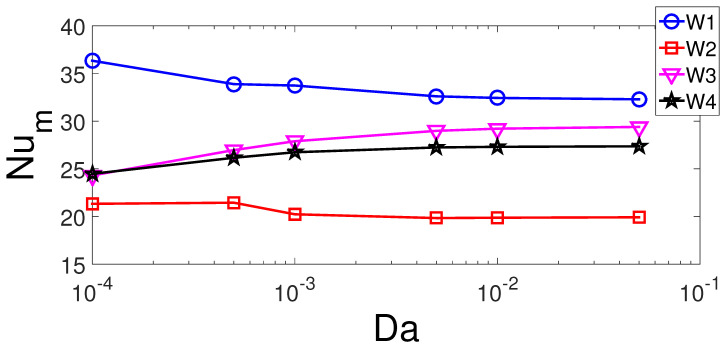
Average Nu variation of individual hot walls with varying values of porous layer permeability (Re = 500, Ha = 15, γ=45°, t${}_{p}$ = 0.3 H, y${}_{p}$ = 0.3 H, b = 0.25 H).

The effects of permeability of the porous region (D2) on the FP and TP variations are shown in Figure 6. The vortex size below the inlet increases with higher permeability of the curved porous layer while the core size moves toward the bottom wall. There is also some slight variations of the upper corner vortex of the VC with varying Darcy numbers. There are 12.5% and 6.7% increases in the average Nu for hot walls W1 and W2, respectively, when highest and lowest permeability cases are compared (Figure 7). For the lowest permeability of the porous layer, it deflects more fluid flow toward the left and bottom walls which reduces the separated flow region below the inlet. However, for right and top hot walls, the average Nu rises with higher permeability of the porous layer which are 21% and 12.5% for hot walls W3 and W4.

**Figure 8:**
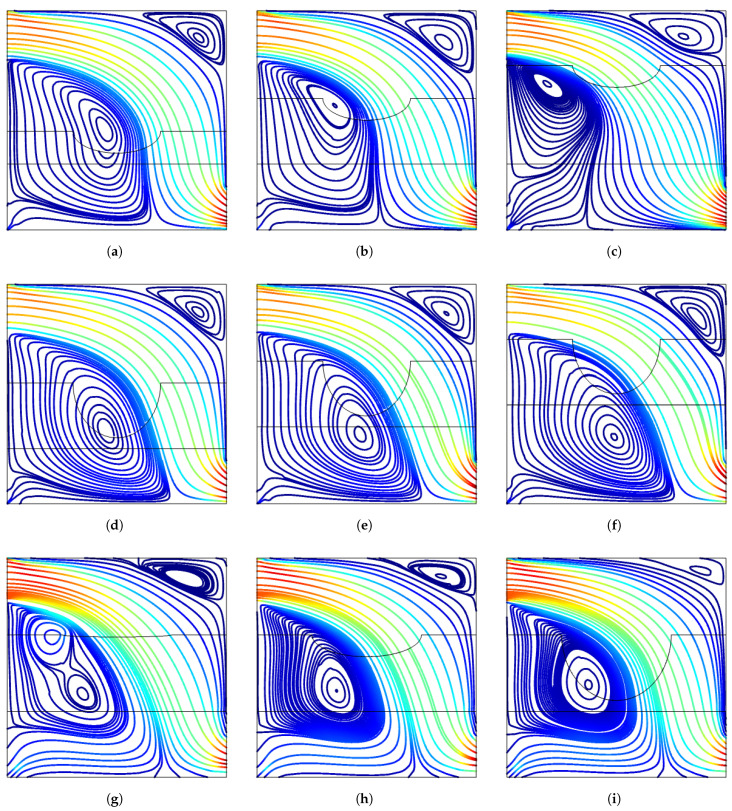
Impacts of curved porous layer partition geometric parameters on the streamline distributions (Re = 500, Ha = 15, γ=45°, Da=$5\times 10^{-2}$). (**a**) t${}_{p}=0.1\;H$, (**b**) t${}_{p}=0.3\;H$, (**c**) t${}_{p}=0.45\;H$, (**d**) y${}_{p}=0.25\;H$, (**e**) y${}_{p}=0.3\;H$, (**f**) y${}_{p}=0.45\;H$, (**g**) b = 0, (**h**) b = 0.1 H, (**i**) b = 0.3 H.

**Figure 9:**
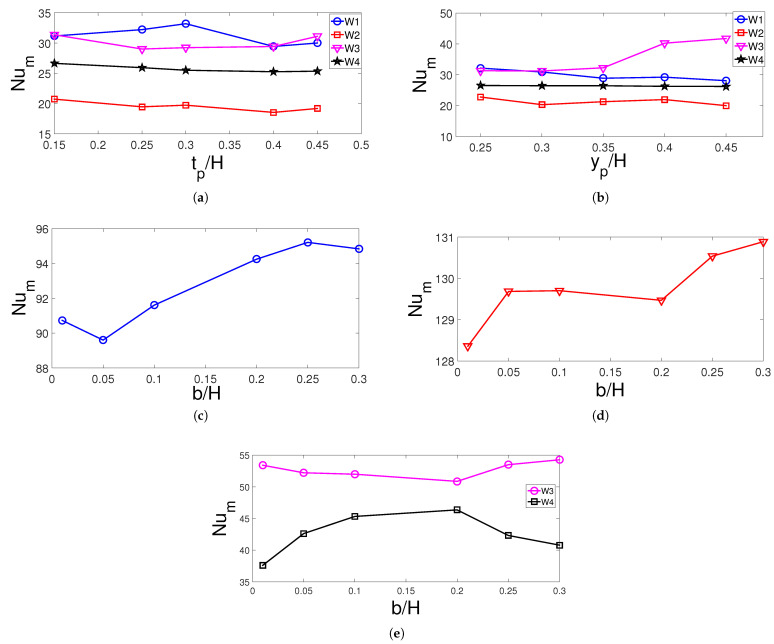
Average Nu variation of hot walls for varying geometric parameters of the curved porous layer partition (Re = 500, Ha = 15, γ=45°, Da = $5\times 10^{-2}$). (**a**) W1, W2, W3 and W4, (**b**) W1, W2, W3 and W4, (**c**) W1, (**d**) W2, and (**e**) W3 and W4.

Impacts of porous layer geometrical parameters on the variation of FP are shown in Figure 8. As the height of the porous layer is increased, the vortex below the inlet reduces in size, while the core center moves toward the inlet and the porous layer effects become important. The upper corner vortex elongates slightly with higher height of the porous layer. The impacts of porous layer location on FP distribution are slight while effects are more profound for changing the curved size of the layer. Two core centers are seen in the vortex below the inlet for case without curvature of the layer while upper corner vortices are also slightly affected with varying *b* values. The highest variation in the average Nu for varying porous layer height is obtained for left hot wall W1 which is attributed to the redistribution of the vortex below inlet port. It increases by about 6% from $t_{p}=0.15\;H$ to $t_{p}=0.3\;H$ and then is is reduced by about 9% from $t_{p}=0.3\;H$ to $t_{p}=0.45\;H$. For other walls, the variations of average Nu are below 4% (Figure 9). As the location of porous zone changes, the highest impact on average Nu is seen for hot wall W3 above the exit port and the variation is about 32.5% when comparing the values between lowest and highest $y_{p}$. For hot wall W1, the average Nu reduces and the highest variation with $y_{p}$ is 12.5%. As the curvature of the porous layer increases, there is only 4.5% and 2.5% variation of the average Nu for hot walls W1 and W2, while the impacts become effective for walls W3 and W4. The lowest average Nu is obtained at b=0.2H for hot wall W3 while for this case, the average Nu is highest for wall W4. The amount of variations in the average Nu is 8% for wall W3 and 24% for wall W4.

**Figure 10:**
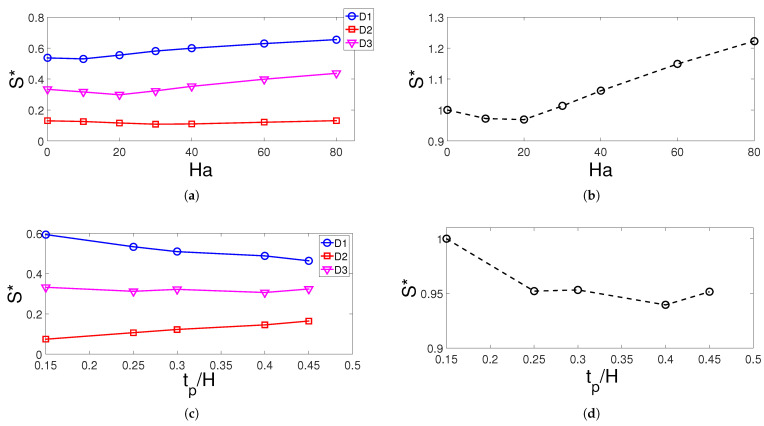
Normalized entropy generation (S*) variation of individual domains and all domains with respect to changes in MF strength (**a**,**b**) and porous layer thickness (**c**,**d**) (Re = 500, γ=45°, Da=$5\times 10^{-2}$, y${}_{p}$ = 0.3 H, b = 0.25 H).

**Figure 11:**
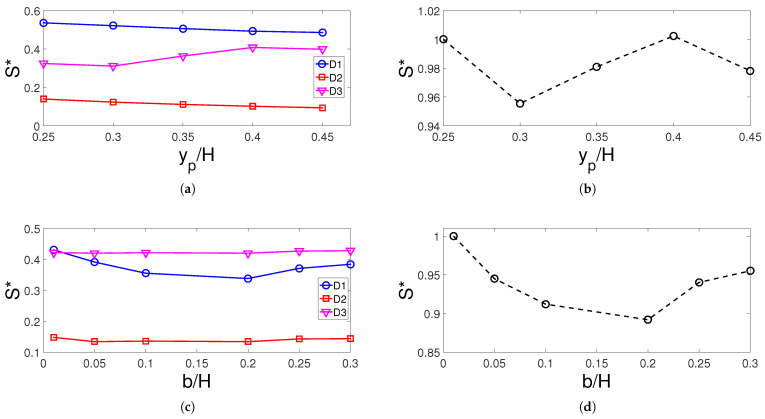
Normalized entropy generation (S*) variation of individual domains and all domains with respect to changes in porous layer location (**a**,**b**) and curvature size (**c**,**d**) (Re = 500, Ha = 15, γ=45°, Da = $5\times 10^{-2}$, t${}_{p}$ = 0.3 H).

The entropy generation (EG) studies are performed for the individual domain and whole domain of the computational model. Effects of MF strength on the variation of EG of domains D1, D2, and D3 and whole domain are shown in Figure 10a,b. The EG is highest for domain D1 and the values increase for Ha number higher than 20. This could be attributed to the higher irreversibility in heat transfer with higher MF strength. When normalized EG (S*) values are compared, there is almost 22% increase for the cases with and without MF effects. The height of the porous layer resulted in reduction of EG for domain D1 which has the highest contribution to the overall normalized EG. There is a 5% reduction when cases at $t_{p}=0.15\;H$ and $t_{p}=0.3\;H$ are compared. The location of the curved porous layer has highest impact on the variation of normalized EG of the domain D3 while the lowest EG is attained at $y_{p}=0.3\;H$ when all domains are considered which is again may be attributed to the lower irreversibility of heat transfer at this configuration. The size of the elliptic curvature has the highest impact on the normalized EG for domain D1 while the overall EG reduces until b=0.2H by about 10% and then increases by about 5% at b=0.3H (Figure 11).

## 4. Conclusions

Impacts of a curved porous layer and MF on the forced CHT and entropy generation in a vented cavity are numerically explored. As the nanofluid velocity rises, the recirculation zone size below the inlet and vortex near the upper corner increases. The impact of Re number on the average Nu increment is significant for hot vertical walls. MF suppresses the vortices within the VC. The MF strength rises the average Nu of hot walls W1, W2, and W4 for Hartmann number above 20, while increment amounts are 47%, 38%, and 38%. However, EG also rises with highest MF strength and 22% increment is obtained when cases with and without MF effects are compared. The presence of the curved porous layer affects the CHT and EG of the vented cavity. As the permeability of the porous layer decreases, more fluid flows toward the walls below the inlet and bottom wall, resulting in CHT increment while the impact seems reverse for right and top hot walls. The increment of average Nu for wall below the inlet is 12.5% with lowest and highest permeability while variation is 21% for right vertical wall. The highest impact of varying height of the porous layer is obtained for wall below inlet port while highest variation in the average Nu is 9%. There is 5% reduction in the total EG when lowest and highest height of the porous layer cases are compared. The porous layer vertical position resulted in change of average Nu of 12.5% for left hot wall and 32.5% for right vertical wall. The lowest EG when varying location of porous layer is observed at $y_{p}=0.3\;H$ for all domains which is attributed to the heat transfer irreversibility. The highest impact of the curvature of the porous layer on the average Nu is attained for top wall which is 24%. However, the total EG reduces with higher radius of the elliptic curvature and lowest value of the total EG is obtained at b=0.2H.

## Figures and Tables

**Table 1:** Thermophysical properties [24].

Property Name	Water	Ag	MgO
k(W/mK)	0.61	45	0.62
μ(kg/ms)	8.55 × 10${}^{-4}$	-	-
$\;c_{p}\;\left(J/\mathrm{kg}\;K\right)$	4179	235	955
$\rho \;(\mathrm{kg}/m^{3})$	997.1	10,500	3560

**Table 2:** Average Nu comparisons in a differentially heated porous cavity.

	Ra = 100	Ra = 1000
Ref. in [37]	3.160	14.060
Ref. in [38]	3.002	13.726
Ref. in [39]	3.115	13.667
Present code	3.112	13.711

**Table 3:** Average Nu comparison for convective heat transfer (CHT) under the effects of magnetic field at Ha = 30 with different Rayleigh numbers.

	Ra = 10${}^{3}$	Ra = 10${}^{5}$
Current study	1.032	3.206
Ref. in [36]	1.002	3.150

## Abbreviations/Nomenclature

*a, b*	elliptic curvature radii
D	domain
Da	Darcy number
Ha	Hartmann number
*h*	local heat transfer coefficient
*k*	thermal conductivity
*n*	unit normal vector
Nu${}_{s}$	local Nusselt number
Nu${}_{m}$	average Nusselt number
*p*	pressure
Pr	Prandtl number
*R*	normalized residual
*r*	neck curvature
Re	Reynolds number
*S* ${}_{g}$	entropy generation
*t* ${}_{p}$	porous layer height
*T*	temperature
*u, v*	x-y velocity components
*w*	port size
*W*	hot wall
*x, y*	Cartesian coordinates
*y* ${}_{p}$	porous layer location
**Greek Characters**	
α	thermal diffusivity
ϕ	solid volume fraction
ν	kinematic viscosity
θ	non-dimensional temperature
ρ	density of the fluid
Ψ	scalar transport variable
**Subscripts**	
*c*	cold
*h*	hot
*m*	average
*nf*	nanofluid
*p*	solid particle
